# Potential of Compounds Originating from the Nature to Act in Hepatocellular Carcinoma Therapy by Targeting the Tumor Immunosuppressive Microenvironment: A Review

**DOI:** 10.3390/molecules28010195

**Published:** 2022-12-26

**Authors:** Yunheng Li, Hui Li, Qiaojun He, Xiaochun Yang

**Affiliations:** 1 Center for Drug Safety Evaluation and Research, College of Pharmaceutical Sciences, Zhejiang University, Hangzhou 310058, China; 2 Innovation Institute for Artificial Intelligence in Medicine of Zhejiang University, College of Pharmaceutical Sciences, Zhejiang University, Hangzhou 310058, China; 3 Shandong (Linyi) Institute of Modern Agriculture, Zhejiang University, Linyi 276000, China; 4 Hangzhou Institute of Innovative Medicine, College of Pharmaceutical Sciences, Zhejiang University, Hangzhou 310058, China

**Keywords:** hepatocellular carcinoma, immunotherapy, immune suppression, TME, natural products

## Abstract

Hepatocellular carcinoma (HCC), the most prevalent subtype of liver cancer, is the second main reason for cancer-related deaths worldwide. In recent decades, sufficient evidence supported that immunotherapy was a safe and effective treatment option for HCC. However, tolerance and frequent recurrence and metastasis occurred in patients after immunotherapy due to the complicated crosstalk in the tumor immunosuppressive microenvironment (TIME) in HCC. Therefore, elucidating the TIME in HCC and finding novel modulators to target TIME for attenuating immune suppression is critical to optimize immunotherapy. Recently, studies have shown the potentially immunoregulatory activities of natural compounds, characterized by multiple targets and pathways and low toxicity. In this review, we concluded the unique role of TIME in HCC. Moreover, we summarized evidence that supports the hypothesis of natural compounds to target TIME to improve immunotherapy. Furthermore, we discussed the comprehensive mechanisms of these natural compounds in the immunotherapy of HCC. Accordingly, we present a well-grounded review of the naturally occurring compounds in cancer immunotherapy, expecting to shed new light on discovering novel anti-HCC immunomodulatory drugs from natural sources.

## 1. Introduction

As the most common type of liver cancer and the second main reason for cancer-related deaths worldwide, hepatocellular carcinoma (HCC) is a major global health challenge [1]. With the worldwide epidemic of obesity, the incidence of nonalcoholic fatty liver disease (NAFLD), and subsequent HCC, are rising markedly. By 2025, HCC is anticipated to affect more than one million individuals worldwide [2]. The treatment options for the early stage of HCC include surgical resection, radiofrequency ablation, transarterial chemoembolization, and liver transplantation. However, many patients have progressed to advanced HCC already in the first diagnosis [3]. For patients with advanced HCC, palliative systemic treatment with sorafenib was once the only choice to relieve symptoms [4]. Unfortunately, the number of patients who actually benefited from sorafenib was minimal due to highly variable clinical responses and side effects [5]. In recent years, immunotherapies, such as immune checkpoint inhibitors (ICIs), have revolutionized the systemic management of advanced HCC [6]. The application of monoclonal antibodies that targeted either the programmed cell death 1 (PD-1)/programmed cell death ligand 1 (PD-L1) or cytotoxic T-lymphocyte-associated protein 4 (CTLA-4) pathway has shown superior clinical outcomes in HCC [2]. Nevertheless, the immunotherapeutic approaches could only induce a durable response in a small subset of HCC patients as the immunosuppressive feature of the tumor microenvironment (TME) [2,7]. Thus, strategies for targeting tumor immunosuppressive microenvironment (TIME) to enhance immunotherapy efficacy are still desperately needed.

There is a unique immune microenvironment in the liver tumor tissue, consisting of heterogeneous populations, including cancer cells, infiltrating immune cells, stromal cells, and various other factors [8]. However, the TME is apt to favor immune suppression rather than immunity through multiple mechanisms, ultimately leading to a tumor immune escape [9]. These cells, factors, and consequent biological processes comprise a complex immunosuppressive network in the TME of HCC. The immune suppression can help tumor cells escape from immune recognition and attack, especially from the effector T cells [10].

Natural compounds, identified as compounds isolated or optimized from nature, have shown great potential in tumor immunotherapy due to their ability to multi-targets/pathways regulation [11]. Naturally occurring compounds can affect various immune molecules regulating the TIME of HCC [10,12]. Additionally, they exhibit the advantages of low toxicity, few side effects, and a wide range of supplies. Thus, more and more research suggests that finding a suitable natural compound to alleviate immunosuppression is a potential therapeutic approach to HCC in the future.

Despite a few systematic reviews about the anti-HCC potential of natural products have been published recently in the related field [13,14,15,16], the underlying influence of natural compounds in targeting the TIME for treating HCC still remains poorly understood and there is a need to further comprehensively review the roles of these natural compounds in the TIME of HCC. This paper aims to elaborate on the current knowledge of the TIME in HCC and explore how they work to induce immune suppression in TME. Additionally, we summarize the newly published research about the significant effects of compounds originated from nature on remodeling TIME of HCC and describe their mechanisms. In this review, we aim to illustrate the crucial role of natural compounds in the field of HCC immunotherapy and provide new insights for future studies.

## 2. The Immunosuppressive Network in the HCC Microenvironment

For current HCC immunotherapies, their efficiencies mainly depend on amplifying and broadening T lymphocyte response [10]. Unfortunately, the immunosuppressive feature of TME weakens the efficiencies of T lymphocyte response. Yang Y. has concluded that TIME mechanisms included abrogating the antigen processing and presentation (APP), recruiting immunosuppressive cells and factors, and up-regulating co-inhibitory factors to weaken the immune response [17].

**Figure 1:**
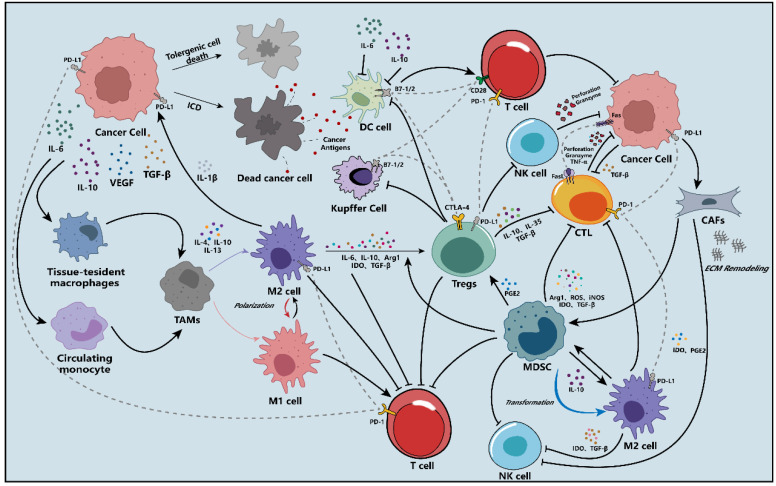
A schematic overview of the mechanisms of the tumor immunosuppressive microenvironment (TIME) in the hepatocellular carcinoma (HCC).

The detailed schematic diagram is shown in Figure 1.

### 2.1. The Deficient Antigen Processing and Presentation Process

To initiate the innate immune cycle, the antigen released from HCC cells must first be recognized and presented to effector lymphocytes, namely APP.

The immunosuppressive secretion of TIME inhibits the activation and function of antigen-presenting cells (APCs), which include dendritic cells (DCs), Kupffer cells (KCs), non-myeloid cells (such as liver sinusoidal endothelial cells, hepatic stellate cells) [18]. The proteasome in cancer cells is inhibited by the TIME, which means that the supplement of antigenic peptides for binding to MHC I is damaged [19]. Kitamura et al. have demonstrated that interleukin (IL)-6 can induce immune suppression by downregulating the expression of MHC II on the DCs [20]. Additionally, IL-6 and IL-10 have been confirmed to activate the transcription factor STAT3, meaning the dysfunction of DCs.

### 2.2. Immunosuppressive Cells and Molecules in the TME of HCC

#### 2.2.1. Regulatory T Cells

Regulatory T cells (Tregs) are a unique subtype of T cells, which can inhibit the immune response by either contact-dependent or cytokine-dependent mechanism in the TIME. Previous research has shown that Tregs were aberrantly increased in the peripheral blood and TME of patients with HCC than normal individuals [21,22]. It has been proved that the accumulation of Tregs in the TIME was associated with a decrease in the CD8+ cytotoxic T lymphocytes (CTLs) proliferation [23]. Tregs also produce immunosuppressive cytokines to inhibit immune response, such as transforming growth factor (TGF)-β, IL-10, and IL-35 [24,25]. Contact-dependent inhibition is another immunosuppressive manner of Tregs. Compared with CD28, constitutive CTLA-4 on the surfaces of Tregs preferentially binds to B7 on the DCs, causing the internalization or sequestration of B7 molecules to impede activation and proliferation of effector T cells [26,27].

#### 2.2.2. Tumor-Associated Macrophages

Tumor-associated macrophages (TAMs) are the most abundant infiltrative immune cells found in the TME. They are broadly divided into two subtypes, M1-like and M2-like TAMs [28]. Different from the M1-like TAMs, M2-like cells promote the development of immune suppression by producing immunosuppressive factors, such as arginase 1 (Arg1), indoleamine 2,3-dioxygenase (IDO), IL-10, IL-6, and TGF-β [2]. Moreover, Tregs can be induced to infiltrate the TME by cytokines secreted from the M2-like TAMs [7]. Under persistent hypoxia of the TME, the M2-like TAMs can release IL-1β induced by necrotic debris of tumor cells, which promotes the expression of PD-L1 and facilitates the recruitment and infiltration of MDSCs to the immune microenvironment of HCC [29]. It has been reported that the amount of M2-like TAMs is inversely proportional to the prognosis of HCC patients [30].

#### 2.2.3. Marrow-Derived Suppressor Cells

Increasing evidence has demonstrated that marrow-derived suppressor cells (MDSCs) are clinically linked with the poor prognosis of HCC therapy by inhibiting the immune effector cells or inducing immunosuppressive cells [31]. As the hub node of immunosuppressive cells in the TIME, MDSCs are characterized by heterogeneity and plasticity [32]. They can transform into other immunosuppressive cells to damage the immune response [33]. Moreover, they can also secrete various immunosuppressive products to impair innate immune response, such as ARG1, reactive oxygen species (ROS), inducible nitric oxide synthase (iNOS), IDO, and TGF-β [34]. Dysregulation of these immunosuppressive cytokines may bias the balance towards immunosuppression.

#### 2.2.4. Cancer-Associated Fibroblasts

Cancer-associated fibroblasts (CAFs) are a component of the TIME transformed from fibroblasts and epithelial cells. They induce MDSCs generation by activating the IL6/STAT3 axis. Moreover, IDO and prostaglandin E2 (PGE2) released from the CAFs attenuate the cytotoxicity of NK cells against tumor cells [35]. Besides, CAFs build a physical barrier to prevent effector T cells infiltration in tumor tissue via remolding extracellular matrix (ECM) network (composed of collagen fibers and glycoproteins), contributing to the formation of TIME in the HCC [36].

### 2.3. Immune Checkpoints in the TME of HCC

In the TIME of HCC, tumor cells can induce the exhaustion of effector T cells by tuning the expression of immune checkpoints to dampen the immune attacks. The binding of inhibitory ligands and receptors down-regulates the immune response mediated by effector T cells [37,38]. CTLA-4, a homolog of CD28, mainly inhibits immune response via two aspects: 1) competitively binding to the B7 on APCs, 2) at the priming phase, delivering an inhibitory signal to T cells in lymph nodes to terminate the activation of effector T cells [39]. Unlike CTLA-4, the PD-1/PD-L1 pathway primarily mediates CTLs migration, proliferation, and cytotoxic secretion to suppress immune response [40].

## 3. The Natural Compounds Attenuate Immune Suppress by Targeting the TIME of HCC

In recent years, significant concerns have arisen about natural products due to an increasing number of studies demonstrating that they can recode the TIME for HCC patients with their systematic regulation property [10,12]. As the studies shown, natural compounds influence the immune system in the TME by following reasons: (1) inducing immunogenic cells death (ICD) to enhance the immunogenicity, (2) inhibiting the production of immunosuppressive cells and cytokines, (3) reducing the expression of immune checkpoints, (4) altering environmental characteristics that induce immune suppression.

All natural compounds which apply in the treatment of HCC and their mechanisms are presented in Table 1.

### 3.1. Polyphenols

Polyphenols are defined as complex substances with two or more phenolic rings joined together. Current evidence strongly supports polyphenols’ contribution to alleviating immune suppression in the TIME of HCC [41,42]. The following work has classified the polyphenols into flavonoids and non-flavonoids, and their specific mechanisms have been thoroughly described.

#### 3.1.1. Flavonoids

Flavonoids, having a basic structural unit of 2-phenylchromone, are further classified into various subclasses: flavonols, flavones, flavanols, anthocyanidins, flavanones, and isoflavones [12].

##### Icaritin

Icaritin is a primary bioactive ingredient derived from the traditional Chinese herb Epimedium (Table 1). ICD of cancer cells, as a particular form of apoptosis, allowed hosts to recognize their dead cell-related antigens, activating a corresponding immune response. Yu Z et al. reported that icaritin could increase the colocalization of mitochondria and autophagosome [43]. Subsequently, both mitophagy and apoptosis of HCC cells would be significantly enhanced [43]. Moreover, to further explore whether this apoptosis of HCC cells induced by icaritin could activate the ICD, they evaluated the levels of chaperone calreticulin (CRT) and high mobility group protein B1 (HMGB1), which were two released damage-associated molecular patterns (DAMPs) followed by the ICD. As expected, the exposure of CRT on the cell surface and the cell death-associated release of the HMGB1 was observed in the presence of icaritin, suggesting that icaritin could enhance the ICD by inducing the death of HCC cells [43]. As a result, the poor immunogenicity was improved by the icaritin-induced ICD effect [43].

Additionally, icaritin could modulate the constitution of immunosuppressive cells and cytokines in the TIME, which favored the immune response for killing HCC tumor cells [44]. Zhou J et al. have indicated that the application of icaritin decreased the number of MDSCs in the spleen of HCC tumor-bearing mice [42]. Moreover, icaritin could facilitate the differentiation of MDSCs into DCs and macrophages [42]. With the administration of icaritin, the production of immunosuppressive cytokines IL-6/10 was also decreased in a dose-dependent manner. Furthermore, the restoration of interferon (IFN)-γ released from CD8+ T cells and a reduction of NO and ROS mediated by MDSCs were also observed in vivo when treated with icaritin. Pro-inflammatory proteins S100A8 and S100A9, as the crucial mediators of expansion and differentiation of MDSCs in HCC, were the major targets of icaritin for improving the TIME [45]. Icaritin decreased the amount of MDSCs and relative factors by downregulating the expression of the S100A8/9 and phosphorylation of STAT3 and Akt in MDSCs, thus interfering with the immune suppressive network of HCC [42]. Moreover, icaritin inhibited splenic extramedullary hematopoiesis (EMH), an approach for accumulating MDSCs in the tumor tissue [46]. At the same time as the reduced frequency of MDSCs infiltration, icaritin inhibited PD-L1 expression on the surfaces of MDSCs and neutrophils. Mechanistically, icaritin was selectively bound to IKK-α to inhibit IKK complex formation from downregulating the NF-κB signaling pathway in monocytes [47,48]. Besides, the enhancive CD8+ T cells and effector memory T-cells were also coordinated with icaritin’s immune anti-cancer process [47]. Strikingly, icaritin has been investigated in the late stage of phase III trials (NCT03236636 and NCT03236649) in China for the treatment of advanced HCC as a single agent; in addition, it exhibited favorable safety, tolerability, and preferable overall survival rate than sorafenib in phase II/III [44,49].

##### Chrysin

As a bioactive flavonoid, chrysin was found in propolis and numerous plants (Table 1). Recently, several studies have reported that chrysin can enhance the anti-tumor response of CTLs by activating APCs or decreasing the levels of IL-1β and IL-6 [50,51]. Given the pivotal role of PD-L1 in negatively influencing the immune response of effector T cells, HCC cells were treated with chrysin to investigate whether chrysin could impair the PD-1/PD-L1 axis for expanding the anti-cancer immunity potency of T cells [41]. As expected, Rong W et al. demonstrated that chrysin strongly suppressed the activation of the IFN-γ-induced JAK/STAT3/IRF-1 axis, which was associated with the expression of PD-L1 [41,52]. Due to the reduced expression of PD-L1 on the surfaces of tumor cells, the increasing proliferation and infiltration of CTLs in the TME were detected. Moreover, NF-κB, a transcription factor directly regulating PD-L1 transcription, was also involved in the chrysin-mediated alleviation of immune suppression. Chrysin could inhibit the NF-κB activation by preventing phosphorylation. Meanwhile, the production of IL-2, the inducer of CD4+/CD8+ T cells, was enhanced evidently in the TME when treated with chrysin. And the higher proliferation of CTLs in the TME was illustrated remarkably [41]. With the development of chrysin targeting the immunosuppression, its derivative 8-bromo-7-methoxychrysin (BrMC) was also added to the conditional medium of TAMs to evaluate if BrMC could influence immunosuppressive cells for improving the immunity of HCC. BrMC inhibited the activation of NF-κB and reversed the M2 polarization of the TAMs; apparently, the secretions of the TAMs were also influenced [53].

##### Luteolin

Luteolin is a natural anti-cancer product in various vegetables and herbs, such as pepper, thyme, and honeysuckle (Table 1). In 2005, the application of luteolin in the treatment of HCC was first reported by Chang J et al. [54]. Later, Kaneko M et al. reported that luteolin could suppress the production of TNF-α and TNF-α-induced signaling [55]. They concluded that attenuating the accumulation of lipid rafts on the surfaces of immune cells (such as macrophages, B cells, and T cells), which were essential for the signaling transduction, was the critical mechanism of luteolin [55]. Moreover, the reduction of immunosuppressive cytokine IL-6 was also observed when the HCC mice were administrated with luteolin [55]. M2-like TAMs, associated with HCC progression and metastasis, played essential roles in the TIME. Under the hypoxia condition induced by cobalt chloride (CoCl_2_), luteolin decreased the levels of vascular endothelial growth factor (VEGF) and matrix metalloproteinase (MMP)-9 via suppressing the activation of hypoxia-inducible factor (HIF)-1 and STAT3 signaling pathway, particularly within the M2-like TAMs [56]. As the intracellular pathways of M2-like TAMs interfered, their immunosuppressive characteristics were also altered by luteolin [56].

Additionally, luteolin suppressed the activation of the YAP pathway, which mediated the polarization of the M2-like TAMs [57,58]. Accompanied by increasing M1-like TAMs, the reduced number of exhausted CD8+ T cells was detected [57,58]. Together with the results, the conclusion was proposed that luteolin inhibited tumor growth by reversing the TIME in HCC. Up to now, the achievements about luteolin’s immune efficacy on HCC are still much less reported. However, the reduced expression of PD-L1 and less infiltration of MDSCs and cytokines/chemokines in luteolin treatment encouraged researchers to explore the anti-HCC effect of luteolin [59,60]. Moreover, the effects of luteolin on enhancing immune response to attack tumor cells by alleviating immunosuppression have been demonstrated in many other cancer types, such as prostate cancer and lung cancer [59,60].

##### Apigenin

Apigenin, an edible plant-derived flavonoid, attracted lots of attention because of its anti-cancer effect with little side effect (Table 1) [59,61]. NK cells, a group of specialized immune effector cells, played critical roles in immune activation against tumors [62]. However, their efficacy against HCC was still poor due to the significant restriction of the immunosuppressive TME. The previous studies showed that the cytotoxic effects of NK cells on the tumor cells decreased dramatically when the level of HIF-1α was high in the TME [63]. However, the secretion of granzyme B (GrzB) from NK cells, which could enter HCC cells to induce their apoptosis, was increased by apigenin. Besides, apigenin could enhance the expression of death ligand CD95L on the surfaces of NK cells [64]. The binding of CD95L/CD95 could trigger more apoptosis of tumor cells via facilitating the expression of caspase-8 and caspase-10 [64]. As the main ingredient of the Dahuang Zhechong pill (DHZCP), apigenin inhibited the origination of Tregs via regulating the TGF-β/Smad pathway [65]. The balance of Treg/ type 1 T helper (Th1) contributed to the reduction of volume and weight of the tumor [65].

##### Quercetin

Quercetin, containing a basic scaffold of phenylbenzopyrone structure (C6-C3-C6), is a typical flavonoid presenting in the plant kingdom (Table 1) [66]. Quercetin showed dominant inhibitory activity towards immunoregulatory proteins [67]. Moreover, quercetin decreased IL-6/IL-1β in the rats with early HCC for initiating the immune response to kill tumor cells [68]. Besides, some studies suggested that quercetin avoided immune escapes of tumor cells by inhibiting the EMT [69]. Mechanistically, quercetin reversed the EMT process by inactivating the JAK2 and STAT3 [69]. Pentamethyl-quercetin, one of the polymethoxylated flavones, demonstrated that it could downregulate the expression of PD-L1 in HepG2 cells (Table 1) [70]. Compared with the control mice bearing tumor, obese HCC-bearing mice showed higher expression of PD-L1 due to more secretion of IFN-γ from adipocytes [70]. Therefore, Li Z et al. investigated whether pentamethyl-quercetin could decrease the PD-L1 expression level for retarding the development of HCC in obese mice [70]. The results suggested that pentamethyl-quercetin abrogated IFN-γ signaling, thus inhibiting the expression of PD-L1 [70]. Furthermore, PD-L1-induced apoptosis of CD8+ T cells was also reduced by pentamethyl-quercetin [70,71].

#### 3.1.2. Nonflavonoids

Non-flavonoid molecules are subclassified into five types of polyphenols: phenolic, hydroxycinnamic, lignans, stilbenes, and tannins [72].

##### Curcumin

Curcumin is the main bioactive ingredient of Curcuma longa L., a perennial herb widely cultivated in tropical and subtropical areas (Table 1). Although it showed low bioavailability in many clinical trials, its hepatoprotective effect could not be ignored [73]. Its inhibition of immunosuppressive cells and factors, alleviation of hypoxia-induced immunosuppression, and enhanced population of CTLs, have been widely investigated in previous tumor immunotherapies [74]. Bhattacharyya S et al. demonstrated that tumor progression was associated with the loss population of effector T cells in circulation, which the administration of curcumin could restore [75]. Moreover, more CD4+ and CD8+ T cells infiltrated the tumor site and contributed to the immune-mediated death of tumor cells after treatment with curcumin. Intriguingly, the decreased number of memory T cells in the peripheral circulation of HCC could be reversed to normal level by curcumin [75]. Additionally, the augmented population of CD4+CD25+FoxP3+ Tregs and levels of immunosuppressive cytokines, TGF-β and IL-10, were prevented by curcumin [75,76]. Man S et al. illustrated that curcumin could enhance host immune response via downregulating the activation of IL-6/JAK/STAT3 and IL-β/NF-κB pathways, which involved attenuating immunosuppression [77]. EF24, a novel curcumin analog with greater bioavailability but no increased toxicity, was discovered by Adams BK et al. (Table 1) [78]. Furthermore, Thomas SL et al. reported that EF24 inhibited hypoxia via sequestering HIF-1α in the cytoplasm and promoting the degradation of the HIF-1α [78,79]. The improvement of TME inhibited the production and accumulation of MDSCs [78,79].

##### Resveratrol

Resveratrol can be widely found in dietary sources, such as grapes, berries, and peanuts (Table 1). The medicinal value of this anti-HCC natural product was well-documented [80]. Zhang Q et al. proposed that resveratrol decreased the population of CD8+CD122+ Tregs, which were more potent for maintaining immunosuppression than CD4+Foxp3+ Tregs [81]. Meanwhile, the reduced number of M2-like TAMs in the HCC-bearing mice was also observed with the administration of resveratrol [81]. Additionally, resveratrol treatment diminished the production of immunosuppressive cytokines TGF-β1 and IL-10 and elevated anti-tumor cytokines TNF-α and IFN-γ [81]. The researchers dug deeper and concluded that resveratrol attenuated the immunosuppression mainly by inhibiting the activation of STAT3 signaling in the tumor [81]. Notably, resveratrol could also enhance the anti-cancer immune response by increasing the percentage of effector CD8+ T cells in the TME and peripheral lymphoid organs [81]. Hepatic stellate cells (HSCs), one unique type of cells that existed in the liver TIME, facilitated the accumulation of MDSCs via activating the IL-6-induced signaling [82]. However, resveratrol inhibited the receptor proteins expression of IL-6 and CXC-4 by reversing the HSC-induced expression of Gli-1; moreover, the angiogenesis and ROS production in the TME was also abrogated by resveratrol [83].

##### Rosmarinic Acid

Rosmarinic acid is a natural polyphenolic compound extracting from many herbal medicinal species of Boraginaceae and Lamiaceae (Table 1) [84]. The ratio of CD4+/CD8+ T cells and the levels of their characteristically secreted cytokines, IFN-γ and IL-2, were induced to decrease by the tumor cells, while these immunosuppressive issues could be reversed via inhibiting the activation of STAT3 pathway by rosmarinic acid [84]. After rosmarinic acid administration, the hepatic tumor growth in the mice was inhibited significantly without obvious toxicity to the immune organs, suggesting rosmarinic acid’ s therapeutic potential for HCC [84].

**Table 1:** The application of natural products in hepatocellular carcinoma (HCC) immunotherapy.

Classification	Resource	Compound	Structure	Mechanism	Reference
Polyphenols	Epimedium	Icaritin	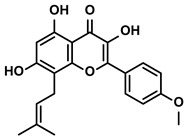	①Enhancing the ICD of tumor cells, improving the poor-immunogenicity ②Reducing the proportion of MDSCs and its secretion IL-6/10, NO, ROS ③Restoring the anti-cancer ability of CTLs ④Inhibiting the expression of PD-L1	[43,44,46,48,85]
	Propolis and numerous plants	Chrysin	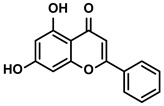	①Impairing the PD-1/PD-L1 axis ②Increasing the production of IL-2 to induce more proliferation of effector T cells	[41]
		BrMC	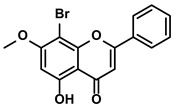	①Reversing the M2-like polarization of TAMs ②Influencing the secretion of immunosuppressive cytokines from TAMs	[53]
	Vegetables, Herbs	Luteolin	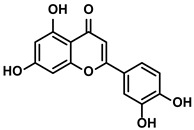	①Reducing the levels of immunosuppressive cytokines, such as IL-6 and VEGF, etc. ②Alleviating the exhaustion of effector T cells ③Reversing the M2-like polarization of TAMs	[54,55,56]
	Plants	Apigenin	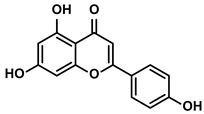	①Recovering the cytotoxicity of NK cells via repairing the connection between tumor cells and NK cells ②Inhibiting the generation of Tregs	[64,86]
	Fruits, Vegetables	Quercetin	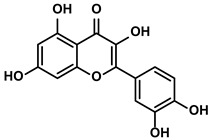	①Impairing the angiogenesis in the TIME, enhancing the immune response	[55,69]
		Pentamethylquercetin	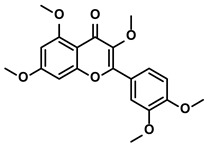	①Reducing the levels of immunosuppressive cytokines IFN-γ ②Reducing the expression of PD-L1 on surfaces of tumor cells	[70]
	Curcuma longa L.	Curcumin	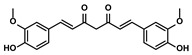	①Increasing the infiltration of effector T cells ②Reducing the proportion of Tregs and the levels of TGF-β and IL-10	[75,87,88]
		EF24	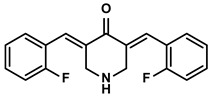	①Improving the hypoxia to relieve the accumulation of MDSCs	[89]
	Veratrum grandiflorum Loes.	Resveratrol	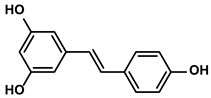	①Decreasing the accumulation of M2-like TAMs and Tregs and the secretion of IL-6/10, TGF-β ②Elevating the percentage of CTLs and immune-related cytokines ③Alleviating the hypoxia and ROS production from tumor cells	[81,83]
	Rosmarinus officinali L.	Rosmarinic acid	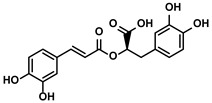	①Increase the levels of IL-2 and IFN-γ and the ratio of CD4+/CD8+ T cells	[84]
Terpenoids	Artemisia annua L.	Artemisinin	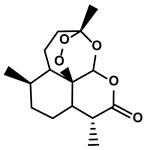	①Inhibiting the accumulation of MDSCs and inducing the repolarization of MDSC ②Reducing the levels of TGF-β, IL-10, and Arg1	[90]
		Artesunate	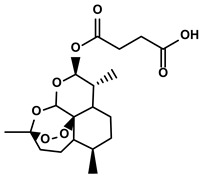	①Inhibiting the release of TGF-β from tumor cells, which impairs the action of T cells ②Facilitating the maturation of DCs to enhance the APP activity ③Increasing the expression of Fas on the surfaces of tumor cells	[91,92]
	Cantharides	Norcantharidin	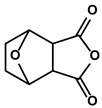	①Stimulating the shift from M2-like TAMs to M1-like TAMs ②Decreasing the CD4+/CD25+ Foxp3 T cells population ③Inhibiting the occurrence of EMT in the TME to easy the infiltration of effector T cells	[93]
	Tanacetum parthenium	Micheliolide	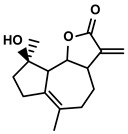	①Inducing ICD-associated DAMPs to trigger the immune response	[94]
	Gardenia jasminoides Ellis	Genipin	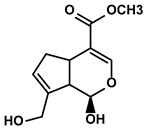	①Impeding the recruitment of TAMs to the TME ②Reducing the levels of protumoral cytokines, IL-6, IL-1β, etc.	[95]
	Curcuma longa L.	Curcumol	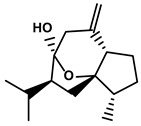	①Inhibiting the expression of PD-L1, recovering the cytotoxicity of CTLs	[96]
Quinones	Angelica sinensis (Oliv.) Diels	Ligustilide	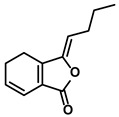	①Inhibiting the macrophage recruitment and M2-like polarization ②Inhibiting the immunosuppressive activity of CAFs	[97,98]
	Salvia miltiorrhiza Bge.	Cryptotanshinone	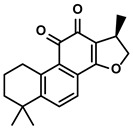	①Reversing the polarization of M2-like TAMs toward M1-like TAMs via influencing metabolism in the TME	[99]
	Rheum palmatum L. and Polygonum cuspidatum Sieb. et Zucc.	Emodin	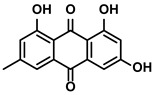	①Inducing the repolarization of M2-like to M1-like macrophage ②Reducing the release of TGF-β	[100,101]
	Plumbago indica L.	Plumbagin	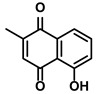	①Inducing ICD to trigger immune response ②Reducing the number of immunosuppressive cells while elevating the number of effector T cells and NK cells	[102]
Alkaloids	Tavernaemontana divaricata and Ervatamia microphylla	Conophylline	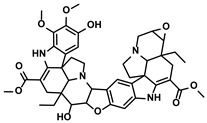	①Inhibiting the immunosuppressive activity of CAFs ②Reducing the levels of immunosuppressive cytokines secreted from CAFs	[103]
	Sophora flavescens Ait.	Matrine	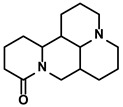	①Facilitating the maturation of DCs to trigger immune response ②Inducing the M1-like polarization of TAMs ③Enhancing the generation of CTLs and related cytokines to kill tumor cells	[104,105]
	Abrus cantoniensis Hance	Abrine	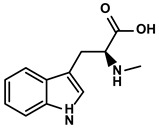	①Inhibiting the expression of PD-L1 ②Inhibiting the apoptosis of effector T cells	[106]
	Nicotiana tabacum	Nicotine	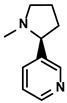	①Accelerating the maturation of DCs and enhancing the APP activity ②Increasing the levels of anti-tumor cytokines, IL-12	[107]
	French lilac (Galega officinalis L.)	Metformin	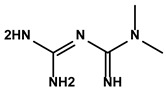	①Decreasing the proportion of MDSCs, M2-like TAMs and Tregs ②Inducing the repolarization of M2-like to M1-like macrophages ③Contributing to the decomposition of ECM to easy the infiltration of effector T cells	[108,109]
Steroids	Bufo bufo gargarizans Cantor	Bufalin	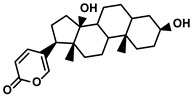	①Influencing the balance of immune cytokines ②Recovering the conventional number of Tregs and MDSCs in the TME ③Reversing the M2-like polarization towards M1-like ④Increasing the Th1/Th2 ratio	[110,111]
Phenylpropanoids	Ruta graveolens L.	4-Methylumbelliferone	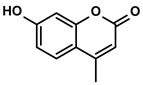	①Inhibiting the synthesis of HA to avoid the angiogenesis and fibrosis in the TIME ②Influencing the polarization of TAMs ③Decreasing the number of MDSCs and Tregs ④Inducing the maturation of DCs to expand antigen presentation ⑤Inhibiting the production of CSCs to make tumor cells more susceptible for immune cells	[112,113,114]
	Cnidium monnieri and Angelica pubescens	Osthole	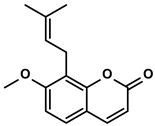	①Decreasing the number of Tregs while elevating the number of effector T cells and their released cytokines	[115,116]
Glycosides	Scutellaria baicalensis Georgi	Baicalin	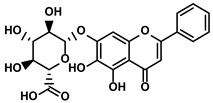	①Repolarizing the M2-like TAMs to M1-like TAMs ②Inhibiting the upregulation of PD-L1 induced by IFN-γ, without influencing the expression of MHC I	[117,118]
	Carthamus tinctorius L.	Safflower yellow	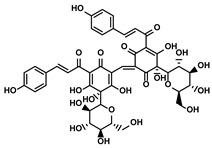	①Promoting the degradation of ECM to let the CTLs infiltrate the TME ②Reducing the number of MDSCs	[119]
		Hydroxyl safflower yellow A	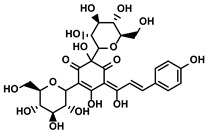	①Decreasing the number of Tregs and Th17	[120]
	Abies georgei	747	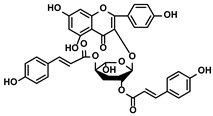	①Impeding CCL2/CCR2 axis to inhibit the recruitment of TAMs to tumor tissue and induce the repolarization of M2-like towards M1-like macrophage ②Benefiting the infiltration of CTLs in the TME	[121]
	Solanum nigrum L.	Solanine	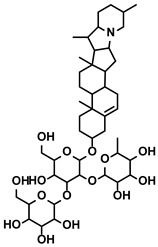	①Blocking the TGF-β signaling pathway, thus alleviating immune suppression ②Lowing the percentage of Tregs in the TME	[122]
		Solamargine	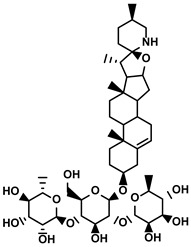	①Initiating the polarization of M1-like TAMs ②Enhancing the recruitment of DCs and inhibiting the infiltration of MDSCs	[123]
	Astragalus membranaceus(Fisch.)Bge.	Astragaloside IV	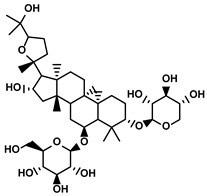	①Inhibiting the polarization of M2-like macrophages	[124]
	Paeonia lactiflora Pall. and Cimicifuga foetida L.	Paeoniflorin	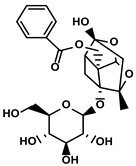	①Inhibiting the expression of PD-L1 ②Increasing the production of immune cytokines to conduct immune response	[125]
	Gastrodia elata Blume	Gastrodin	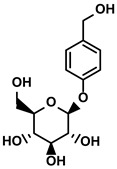	①Inhibit the production of Th2 cells and its cytokines ②Recover the cytotoxicity of CTLs ③Increase the percentage of CD4+ T cells	[126]

### 3.2. Terpenoids

As the most common class of natural products, terpenoids comprise approximately 25,000 compounds and are classified into five subclasses based on structures, including monoterpenoids, sesquiterpenoids, diterpenoids, triterpenoids, and tetraterpenoids [127]. In the following description, we will focus on natural terpenoids that have been reported to inhibit HCC progression via targeting the TIME and enhancing immune response.

#### 3.2.1. Artemisinin

Qinghao, a common natural herb, has been utilized in traditional Chinese medical practice to treat malaria, fever, and chills for over two thousand years. Its active ingredient, artemisinin, was discovered and isolated by Chinese scientist Tu Youyou in August 1972 (Table 1) [128]. Since then, the application of artemisinin has developed worldwide, and more pharmacological activities have been reported, such as anti-cancer and anti-inflammation activities [129]. Artemisinin and its derivates reversed immunosuppression in various cancer models [130,131]. The accumulation of MDSCs in the HCC microenvironment, which inhibited the generation of effector T cells and contributed to the establishment of the protumor TIME, could be blocked markedly by artemisinin [90]. Zhang M et al. demonstrated that artemisinin with a dose of 100 μM could induce the apoptosis of MDSCs and reduce the generation of MDSCs dramatically in vitro [90]. Besides, it triggered the repolarization of MDSCs from M2-like tumor-promoting phenotype towards M1-like antitumoral one via inhibiting the activation of MAPK and PI3K/Akt signaling axes [90]. Notably, the artemisinin-induced alteration of signaling pathways in the MDSCs also influenced the gene expression profiles. By cytokine detection, the downregulation of M2-like signature cytokines, Arg1, IL-10, IL-6, and TGF-β, and upregulation of M1-like signature cytokine, TNF-α, and iNOS, were observed [90]. However, 100μM was a quite high dose as a target-pointed drug in vitro. Therefore, the researchers further explored the anti-HCC effect of artemisinin in Hepa 1-6-bearing mice. The results suggested that only treatment with 50 mg/kg artemisinin significantly decreased the percentages of MDSCs, M-MDSCs, G-MDSCs, and Treg cells while increased the percentages of CD3+/CD4+/CD8+ T cells in vivo [90]. Meanwhile, artemisinin showed no adverse effects on the percentages of DCs, macrophages, and NK cells in the HCC-bearing mice.

With the development of artemisinin, its derivative artesunate was isolated from Artemisia annua (sweet wormwood) (Table 1) [132]. Artesunate showed anti-HCC activity by restricting the immune escapes of tumor cells under the immunosuppressive microenvironment. Artesunate attenuated the effect of immunosuppression on γδ T cells by inhibiting the secretion of TGF-β1 from the HCC cells [92,133]. Additionally, artesunate induced the maturation and activation of DCs, which could initiate the immune cycle [92,133]. Besides, it enhanced the expression of Fas on HCC cells, thus contributing to the Fas/FasL-driven apoptosis of HCC cells [91,92]. In conclusion, the effect of artesunate on targeting immunosuppression was ascribed to the inactivation of IL-6-driven STAT3 [91].

#### 3.2.2. Cantharidin

Cantharidin is a terpenoid isolated from Cantharides and has been used in traditional Chinese medicine for over 2000 years. Previous research confirmed the anti-cancer activity of cantharidin in various cancer models, whether in vitro or in vivo [134,135]. However, its severe toxicity on the digestive and urinary systems was unignorable and restricted its application in further clinical treatment [136]. Thus, norcantharidin, a demethylated form of cantharidin, was synthesized and commonly used for the clinical treatment of primary HCC as a combined chemotherapy drug (Table 1); more importantly, it showed little toxic effects [137,138]. Lu S et al. reported that norcantharidin inhibited tumor-size growth in hepatoma-bearing mice owing to the alteration of the TIME [137]. Specifically, M2-like TAMs were converted to M1-like TAMs, and CD4+/CD25+Foxp3 Tregs were decreased significantly by the norcantharidin [137]. Likewise, norcantharidin reduced the level of Arg1, the marker of M2-like TAMs, while increasing TNF-α, the quality of M1-like TAMs. With deeply mechanism study, the researcher revealed that norcantharidin inhibited the immune suppressive effects of TAMs in the TIME via blocking the STAT3 phosphorylation. Besides, miR-214, a tumor-suppressor involved in repolarizing the M2-like TAMs towards M1-like TAMs, was overexpressed by norcantharidin in the TAMs [137]. Except for the effects we mentioned, norcantharidin also could inhibit the IL6-driven EMT, thus reducing the immune escapes of tumor cells [93,139]. To improve the solubility of norcantharidin, Zhang H et al. invented a glycyrrhetinic acid-modified norcantharidin nanoparticle and examined its anti-HCC ability in vitro and in vivo [140]. As expected, its solubility and targeting improved markedly after being modified.

#### 3.2.3. Micheliolide

Micheliolide is a natural sesquiterpene lactone with a well-known function of improving inflammation-associated cancer (Table 1) [141]. Recently, Xu Z et al. screened out micheliolide as the inhibitor of TrxR via a virtual screening [94]. Thus, micheliolide induced the expression of ICD-associated DAMPs and further accelerated the DCs maturation for activating the CD4+/CD8+ T cells [94]. ICD was characterized by the release and reposition of DAMPs, such as the extracellularly releases of endoplasmic reticulum (ER)-resident chaperone protein CRT, HMGB1, and adenosine triphosphate (ATP); the exposure of heat-shock protein 70/90 (HSP 70/90) on the cell membranes [94]. Namely, a series of molecules acted as danger signals to induce the maturation of DCs and secretion of immune cytokines, which were the keys to activating the immune response. It was well-known that ROS-mediated ER stress contributed to the activation of danger signaling. However, thioredoxin reductase (TrxR), a vital antioxidant enzyme, contributed to scavenging redundant ROS and stopped the further releases of DAMPs induced by ER stress.

#### 3.2.4. Genipin

Genipin is an iridoid derived from geniposide, isolated from Gardenia jasminoides Ellis extract (Table 1). Previous studies have revealed the hepatoprotective and anti-HCC activities of genipin acting as an antioxidant [142]. Therefore, Tan HY et al. explored whether genipin could influence immunosuppression to improve the HCC immunotherapy [95]. Genipin impeded the migration of TAMs into the TME and reduced the expression of TAMs-derived immunosuppressive cytokines, which slowed the HCC progression. They found that genipin downregulated the levels of IL-6 and TNF-α by blocking the inositol-requiring enzyme (IRE)-1α-X-box-binding protein (XBP)-1 axis to inhibit the related genes transcription [95]. Moreover, the researcher discovered that genipin could reduce the expression of iNOS and IL-1β in the M2-like TAMs to inhibit tumor growth [95]. The result suggested that the genipin prevented the formation of a complex composed of the IRE1α, TNF receptor-associated factor (TRAF)-2, and inhibitor of κB kinase (IKK) via inhibiting the expression of IRE1α in the TAMs. As a result, the reduced component of the complex inactivated the NF-κB, attenuating the immunosuppression in the TME [95].

#### 3.2.5. Curcumol

Curcumol, a bioactive sesquiterpenoid, was included in the State Pharmacopoeia Commission of the People’s Republic of China (2005) (Table 1). Curcumol was commonly isolated from the Zingiberaceae family plants, mainly found in China, India, and Peru [143]. It was well-known for its multiple pharmacological activities, such as anti-cancer, anti-fungal, and anti-viral. Recently, its effect on HCC treatment has been reported gradually. Curcumol inhibited the expression of PD-L1 and then restored the cytotoxicity of CTLs for killing the HCC cells [96]. Reportedly, the crosstalk between HIF-1α and STAT3 pathways contributed to the expression of the PD-L1 [96]. However, curcumol blocked the crosstalk via simultaneously inhibiting JAK, mTOR, and MAPK pathways, which were responsible for the phosphorylation of STAT3 and expression of HIF-1α, respectively [96].

### 3.3. Quinones

Quinones were characterized as a group of compounds having intramolecular unsaturated cyclic diketone structure or quickly transforming into such a structure. They could be classified into benzoquinone, naphthoquinone, anthraquinone, and phenanthraquinone [144]. Quinones were named the “privileged structure” in medicinal chemistry because they could bind or interact with various biological receptors to undergo diverse pharmacological activities, such as anti-tumor, anti-bacterial, and anti-viral [144].

#### 3.3.1. Ligustilide

Ligustilide is a benzoquinone derivative isolated from Angelica Sinensis, an herb that has been utilized to improve the weak state of the human body in China for over a thousand years (Table 1) [145]. It was well studied that the circulating monocytes could be recruited into the TME and differentiated into the TAMs. The signals released from tumor cells were beneficial to repolarizing the M1-like to M2-like TAMs, which contributed to interfering with the immune response of effector T cells. Thus, inhibiting the inducible signals released from tumor cells to block the communication between cancer cells and TAMs seemed to be a promising strategy for anti-cancer therapy. Yang J et al. reported that ligustilide reduced the number of TAMs and inhibited the transformation of M1-like to M2-like TAMs [98]. Mechanistically, ligustilide impaired the secretion of the signals from tumor cells via inhibiting the YAP nuclear transcription and expression to inactive the IL-6/STAT3 signaling. Most importantly, the utilization of ligustilide showed little cytotoxicity on normal hepatocytes while preventing the production and accumulation of the immunosuppressive cells and cytokines in the TME of HCC [98].

Moreover, CAF, another primary immunosuppressive cell type in the TIME, was also inhibited by ligustilide in the HCC models [97]. Interestingly, ligustilide dampened the immunosuppressive effect of CAFs without influencing the proliferation of CAFs. The restored T cell recruitment and expansion were observed [97,146]. Reportedly, the activation of the NF-κB pathway could reverse the immunosuppressive function of MDSCs. Inspirited by this theory, Ma J et al. investigated the effect of ligustilide on the NF-κB pathway. They confirmed that ligustilide targeted toll-like receptor (TLR)-4 to trigger the activation of NF-κB signaling, thereby alleviating the immunosuppressive action of CAFs [97].

#### 3.3.2. Cryptotanshinone

Cryptotanshinone was extracted from the root and rhizome of Salvia miltiorrhiza Bge. (Table 1) [147]. Research demonstrated that cryptotanshinone altered the polarization direction of macrophages to an M1-like phenotype by inactivating the NF-κB pathway. With the reduced M2-like TAMs, the exhausted CD8+ T cells gradually disappeared [148,149]. Moreover, the discovery of Jiang T et al., in which cryptotanshinone affected the M1/M2 ratio in the TME of HCC, confirmed the immunoregulatory effects of cryptotanshinone [99]. And they found another cryptotanshinone-mediated mechanism that was associated with reversing the immunosuppression. Metabolism played a significant role in deciding the polarization of macrophages. Unlike the increased glycolysis level in the M1 macrophages, the M2-like metabolic phenotypes were mainly the oxidative phosphorylation of the glucose [150]. Cryptotanshinone could reverse the polarization of macrophages by switching the metabolism from oxidative phosphorylation to glycolysis [99]. Furthermore, this metabolism transformation depended on activating the AMPK signaling [99]. Later, the researcher found that the synergetic action of arsenic trioxide and cryptotanshinone could lead to a more significant inhibition in the progression of HCC and boosted M1-like TAMs. [99].

#### 3.3.3. Emodin

Emodin is a bioactive natural product extracted from the traditional Chinese herbs, Rheum palmatum L. and Polygonum cuspidatum Sieb. et Zucc. (Table 1). It has been confirmed that it has treating effects on liver diseases [100]. During the treatment of HCC, emodin induced the transformation from M2-like to M1-like TAMs while decreasing the TGF-β1 secreting from the M2-like TAMs [101]. In fact, with the further exploration of emodin, Yin J et al. proposed that emodin could upregulate the microRNA-26a expression in the TAMs, which negatively regulated the expression of TGF-β1 [101]. Meanwhile, it inhibited the activation of the Akt pathway associated with the M2-like polarization [101].

#### 3.3.4. Plumbagin

Plumbagin is a quinoid substance isolated from the root of Plumbago zeylanica L. (Table 1). It was utilized as an antimicrobial at the beginning [151]. Later, more and more results suggested that it could treat cancers by influencing multiple pathways, such as Akt/NF-kB, MMP-9, and VEGF [152]. Recently, Han S et al. demonstrated plumbagin’s effect as a potential ICD drug on the HCC therapy for the first time [102]. As the ICD inducer, plumbagin triggered ER stress-associated apoptosis in the HCC cells. The synergistic effect of ICD inducer plumbagin combined with ICD enhancer dihydrotanshinone I enhanced the ICD of HCC cells. The increased expression of DAMPs and tumor antigens reprogramed the immunosuppressive TME in the HCC, embodied in the upregulation of DCs, NK cells, M1-like TAMs, CD8+ cytotoxic/memory T cells, and CD4+ helper/memory T cells [102]. Correspondingly, the downregulation of immunosuppressive cells, such as M2-like TAMs, Tregs, and MDSCs, was also observed when HCC-bearing mice were administrated with the plumbagin and dihydrotanshinone I. Moreover, the plumbagin could increase IFN-γ, IL-12, and TNF-α and decrease IL-4, IL-10, and TGF-β [102].

### 3.4. Alkaloids

Alkaloids are generally defined as a group of compounds containing cyclic structures with at least one basic nitrogen atom. They could be found widely in the plant kingdom, such as the herbs of Leguminosae and Papaveraceae families [153]. Over 3000 natural products have been recognized and classified into the alkaloids [153]. According to previous reports, their biological activities and safety have been confirmed in many other diseases [154]. Therefore, more and more attention has arisen on their effects in treating cancer [155].

#### 3.4.1. Conophylline

Conophylline is a vinca alkaloid first extracted from the leaves of the tropical plant Tavernaemontana divaricate (Table 1). Then it was also isolated from the leaves of Ervatamia microphylla [156]. Given the improvement of liver fibrosis after treatment with conophylline, Kubo N et al. were encouraged to explore the effect of conophylline on the progression of HCC [157]. They found that the application of conophylline could markedly decrease the expression of α-smooth muscle actin (α-SMA) and collagen-1; in other words, it inhibited the activation of HSCs [157]. According to the similarity of activating the HSCs and CAFs, Ishii N et al. hypothesized that conophylline could also inhibit the activation of CAFs, consequently reducing the production of α-SMA, an essential component of the TIME [158]. First, they confirmed this hypothesis in the pancreatic cancer model [158]. Then, as expected, conophylline showed the capability of suppressing the proliferation and function of CAFs in treating the HCC [103]. Meanwhile, conophylline strongly decreased the levels of various immunosuppressive cytokines, such as IL-6/8, C-C motif chemokine ligand (CCL)-2, and CXC chemokine ligand (CXCL)-12, which were secreted from the CAFs [103]. Mechanistically, conophylline inhibited the expression of GPR68, which mainly regulated immune cytokines expression in the CAFs [103].

#### 3.4.2. Matrine

Matrine is characterized as a quinolizidine alkaloid extracted from the root of Sophora flavescens Ait. (Kushen) (Table 1) [159]. Due to its low toxicity and relatively inexpensive raw materials, more attention has been attracted to its immunoregulatory activity in HCC treatment. For example, Zhao B et al. demonstrated that matrine could reverse the M2-like TAMs polarization, thereby impeding immunosuppression-induced lung cancer metastasis [160]. Shortly after that, Zhou N et al. found that matrine could stimulate the phenotype and function maturity of DCs by enhancing autophagy activity in the DCs [105]. This resulted that a more robust antigen presentation would be processed and more Th1 and Th17 immune responses would be activated [105]. Moreover, matrine enhanced the T cells’ proliferation and cytokines secretion, such as IFN-γ and TNF-α. The cytotoxicity of CTLs on the tumor cells was strengthened and stopped tumor growth in the HCC-bearing mice [105]. Besides, matrine reduced the levels of immunosuppressive cytokines, IL-10 and IL-4. Interestingly, matrine, as one of the primary alkaloids in the kushen injection (also known as Yanshu injection), could alter the immunosuppressive polarization of TAMs. This effect mainly depended on targeting tumor necrosis factor receptor superfamily member 1 (TNFR1, also known as TNFRSF1A) by matrine and activating its downstream NF-κB p65 and MAPK p38 signaling cascades in the TAMs for facilitating the M1-like polarization [104]. Moreover, matrine and glycyrrhizin combination therapy alleviated the immunosuppression more effectively. Glycyrrhizin, a triterpene glycoside, has been used as a medically approved drug in treating hepatopathy for over 20 years in China. Moreover, increasing studies about its anti-cancer property have been reported in recent years [161]. The combination therapy group observed more infiltration of CD4+/CD8+ T cells in the TME.

#### 3.4.3. Abrine

Abrine is an indoleamine acids-like alkaloid extracted from Abrus cantoniensis Hance (Table 1) [106]. As a primary alkaloid in available herbs, its potent antioxidative and anti-proliferative properties were revealed many times [162]. Recently, new evidence indicated that abrine could inhibit the growth of HCC cells in vitro and in vivo. Moreover, the expression of PD-L1 was significantly suppressed by abrine via decreasing the expression of lysine acetyltransferase (KAT)-5, a critical enhancer of the transcription and acetylation of the PD-L1 promoter [106]. The reduced proportion of effector T cells induced by PD-L1 overexpression in the TIME was markedly reversed with abrine treatment [106].

#### 3.4.4. Nicotine

Nicotine, a primary alkaloid, was first extracted from the plant Nicotiana tabacum (Solanaceae) in 1828 by German chemists Posselt and Riemann (Table 1) [163]. Regarding the treatment of HCC, Gao FG et al. found that nicotine could significantly enhance the endocytosis of immature DCs (imDCs) and promote the imDCs-dependent T cell priming via activating the PI3K signal pathway [107]. Moreover, nicotine administration achieved a higher level of IL-12 released from DCs. As a result, the activation and differentiation of lymphocytes were enhanced [107]. However, there were also studies suggesting that nicotine could cause M2-like polarization via activating the STAT3 pathway, which was associated with the development of immunosuppression. In conclusion, the effectiveness of nicotine is still needed to be further verified.

#### 3.4.5. Metformin

Metformin is a derivative of galegine extracted from French lilac, so-called Galega officinalis L. (Table 1) [164]. As the most common antidiabetic drug worldwide, metformin has been used in the first-line treatment of clinics for over 60 years. According to [165], the accumulation of MDSCs and M2-like TAMs in the immune microenvironment was prevented by metformin in the NCOA5-deficient mice. Moreover, metformin reduced the HCC incidence in the model group. Mechanistically, metformin inhibited p21 (p21^WAF1/CIP1^)’s aberrant expression [165]. Therefore, the exhausted T cells enriched in the TIME could be relieved after treatment with metformin. Microparticles showed great potential as drug-delivery systems because of their unique identities of messengers between cells. Wei Z et al. invented mannose-modified macrophage-derived microparticles loading metformin (Met@Man-MPs) to target M2-like TAMs for repolarizing to the M1-like TAMs [166]. The increased number of M1-like TAMs effectively inhibited the infiltration of Tregs in the tumor tissue. More importantly, the mannose-modified macrophage-derived microparticles contributed to the degradation of collagen in the ECM, favoring the infiltration of effector T cells into tumor interiors [166].

### 3.5. Steroids

Steroids, a group of compounds with unique four-ring structures, are widely distributed in the plant kingdom [167]. In cancer treatment, steroids have attracted increasing attention from pharmaceutical researchers due to their structural similarity with hormones, a class of substances that intrinsically regulate the lymphoid cells [168]. An increasing number of studies have revealed that steroids could reach the TME and inhibit the immunosuppression [111,123,169].

#### Bufalin

Bufalin, an endogenous cardiotonic steroid, was first isolated from the toad’s skin and parotid venom glands (Table 1) [110]. Bufalin is gaining significant attention in the field of HCC treatment owing to its activities, encompassing reversing various immune resistance, and remodeling the TIME to avoid the exhaustion of effector T cells [111]. Yu Z. et al. demonstrated that bufalin reduced the tumor volume and growth rate by varying the composition of the TIME [111]. The levels of immunostimulatory cytokines, IL-23, IL-12, TNF-α, and IFN-γ, were remarkably increased, while the immunoinhibitory cytokines, IL-4, IL-13, IL-10, and TGF-β, were decreased when HCC-bearing mice were administrated with bufalin. Simultaneously, the predominant infiltration of macrophages and CD4+/CD8+ T cells in the TME was also observed. Notably, the increased number of immunosuppressive cells, Tregs, and MDSCs was not discovered in the bufalin-treated HCC models. With the deep exploration of the mechanism of the bufalin affecting the proliferation and function of effector T cells, they found that bufalin governed polarization of TAMs from the M2-like to the M1-like phenotype and downregulated the M2-associated functional molecules, such as CCL2, CCL22, Arg1, and PD-L1. Then, bufalin-induced upregulation of the M1-like TAMs stimulated the Th1 cell proliferation and Th1-associated anti-tumor cytokines (IL-2 and IFN-γ) accumulation, while decreasing the proportion of Th2 and its pro-tumor cytokines (IL-4 and IL-13) in the TME [111,170]. According to the pivotal role of the NF-κB pathway in the TAM polarization, activating this pathway meant a lot for the TIME composition. As an active hallmark of the NF-κB, the formation of the p65-p50 heterodimer in the nucleus was studied extensively. The researcher suggested that bufalin could promote the transformation of p65 into the nucleus, while decreasing the level of p50 via enhancing the ubiquitination degradation, thereby facilitating the formation of the p65–p50 heterodimer in the nucleus [111]. Different from the previous studies, bufalin activated the NF-κB pathway. However, the M2-like TAMs were not increased, and even more M1-like TAMs were produced by the bufalin, which offset the inhibition of effector T cells caused by the HCC-mediated immunosuppression.

### 3.6. Phenylpropanoids

Phenylpropanoids are defined as a group of aromatic compounds having C6-C3 skeletons. They are subclassified into three classes, simple phenylpropanoids, coumarins, and lignans. Phenylpropanoids exhibited a wide range of biological properties in treating kinds of cancers, especially in the area of reversing immunosuppression to initiate the immune response.

#### 3.6.1. 4-Methylumbelliferone

4-Methylumbelliferone (4-MU), a derivative of coumarin, is primarily found in the plant families of Umbelliferae and Apiaceae (Table 1) [114]. As a natural inhibitor of hyaluronic acid (HA) synthesis, 4-Mu showed unique anticancer activity in the therapy of HCC [112]. The abnormal production of HA boosted the angiogenesis and exacerbated the liver fibrosis to build the TIME. However, this improper synthesis of the HA was abrogated by the 4-MU in the HCC-bearing mice [112,171]. Correspondingly, CXCL12, an immunosuppressive chemokine secreted from the CAFs induced by VEGF, was decreased in the TIME of mice treated with the 4-MU. Moreover, 4-MU could inhibit the abundant production of IL-6 from the HCC cells and KCs. It depended, at least partially, on inhibiting the HA [112]. Inspired by the 4-MU’s effect on targeting the TIME, Mazzolini, G. et al. continued to deeply explore the potential role of 4-MU in the immunotherapy of HCC. The TAMs in the HCC mice were induced to polarize into the M1-like TAMs when the mice were fed with daily 4-MU 200mg/kg [113]. The ratio of iNOS /Arg1 (the markers of M1-like and M2-like TAMs, respectively) was increased fivefold by the 4-MU [172]. Additionally, the increased level of TNF-α while the decreased levels of TGF-β and IL-10 were also achieved with the treatment of 4-MU. Furthermore, after treating with the 4-MU for 72h, the maturation of DCs and their function, such as the antigen presentation and phagocyte, was stimulated significantly. The DC-vaccine culturing with tumor lysates treated with 4-Mu induced a more potent antitumoral effect than the control [113]. In addition to the impact on the DCs, 4-MU also increased the CD3+CD4+/CD8+ T cells but induced a significant decrease of the MDSCs and Tregs levels in the HCC immune microenvironment [113]. On the other hand, 4-MU inhibited the expression of CD47 on the cancer stem cells (CSCs), thereby inducing less aggressive tumor phenotypes and making the HCC cells more susceptible to be recognized by the immune system [113,173].

#### 3.6.2. Osthole

Osthole, a typical compound of linear furanocoumarins, was mainly isolated from medicinal plants, like Cnidium monnieri and Angelica pubescens (Table 1) [174]. Osthole suppressed the growth of HCC tumors both in vivo and in vitro. Moreover, no significant toxicity was observed at its effective dose [116]. Furthermore, osthole inhibited the accumulation of CD4+CD25+Foxp3+ Tregs without immune toxicity in the HCC mice models [116]. With the decreased proportion of immunosuppressive cells in the TME, the increased percentages of effector CD4+ and CD8+ T cells and more secretion of the IL-2 and TNF-α were also detected after administrating with the osthole [116]. Furthermore, CD44+CD62L- T cells, identified as effector/effector memory T cells mainly located at the periphery, were accelerated in the osthole-treated groups [116].

### 3.7. Glycosides

Glycosides, so-called saponin, are compounds composed of glycone, its derivates, and other compounds (such as terpenoids and flavonoids), binding at the anomeric carbons. The introduction of glycone altered the molecules’ characters and improved the targeting precision of molecules, enriching the pharmacological activities.

#### 3.7.1. Flavonoid Glycoside

##### Baicalin

Baicalin was isolated from the herbs of the Scutellaria genus (Table 1) [175]. The remarkable anti-cancer effect of baicalin at both the cellular and creatural level have been demonstrated in the past few years. It was revealed that baicalin could mediate key signaling pathways associated with cancer progression [175]. Tan HY et al. reported that tumor growth was completely inhibited in the HCC mice after orally administrating baicalin at 50 mg/kg for five weeks [118]. The polarization of TAMs towards M1-like phenotypes was induced by baicalin, resulting in the impediment of HCC development. Intriguingly, the polarization caused by baicalin only influenced TAMs and M2-like TAMs without affecting the M1-like TAMs. Instead of regulating the expression of immunosuppressive inducers, baicalin directly facilitated the transformation of M2-like to M1-like TAMs via activating the NF-κB pathway. With further exploration, they found that baicalin initiated the autophagic degradation of the TRAF2, a negative regulator of the RelB/p52 pathway, activating the NF-κB pathway. As a result, baicalin realized the repolarization of M2-like towards the M1-like phenotype [118]. Additionally, baicalin also showed the capability of inhibiting the upregulation of PD-L1 induced by the IFN-γ via mediating the activation of STAT3, thus enhancing the elimination of tumor cells induced by CTLs [117]. As the PD-L1 expression decreased on HCC cell surface, the T cell activity was enhanced, and the level of IL-2 was restored by baicalin. Intriguingly, although the level of IFN-γ was influenced due to the inactivation of STAT3, the expression of MHC I, another molecule induced by the IFN-γ, was not affected, and even increased by the treatment of baicalin [117].

##### Safflower Yellow

Safflower yellow (SY) is the main bioactive ingredient of the Carthamus tinctorius L. (Table 1). Gradually, people discovered that it could enhance the immune response by regulating the immunosuppressive microenvironment in the tumor tissues [119,120]. Fu H et al. reported that the SY improved the immune microenvironment in the HCC-bearing mice [119]. SY inhibited the expression of collagen and MMP-9 in the CAFs to promote the degradation of ECM, thus recovering the infiltration of CD8+ T cells in the TME [36,119]. SY realized the elimination of HCC cells by awakening the innate immune response. Meanwhile, the reduced proportion of MDSCs in the TME was also detected in the HCC mice treated with SY [119]. Later, hydroxyl safflower yellow A (HSYA), an analog of the SY, was also isolated and identified from the Carthamus tinctorius. The research demonstrated that it inhibited the increase of tumor size without side effects [120]. HSYA could simultaneously decrease the number of Tregs and Th17 cells, which had been reported synergistically to promote immunosuppression in the TME [120]. As a result, the progression of HCC was inhibited by the HSYA.

##### Compound 747

Compound 747, an analog of kaempferol, was isolated from Abies georgei by Wang H et al. (Table 1) [121]. After the virtual screening of 43 natural products, 747 showed the strongest affinity and selectivity of binding with CCR2, an important immune-associated target that mediated the macrophage infiltration [121]. The results illustrated that 747 entered the central pocket of the CCR2 and bound with it suitably, inhibiting the CCL2/CCR2 axis activity. Therefore, the recruitment of TAMs in the TME was inhibited, and the shift of M2-like towards M1-like TAMs was achieved [121]. Based on the improved immune microenvironment, the infiltration of CD8+ T cells in the tumor periphery was increased by the 747 treatment, leading to better efficiency of immunotherapy of the HCC [121].

#### 3.7.2. Alkaloid Glycosides

##### Solanine

Solanine, a steroidal alkaloid glycoside, was isolated and identified from Solanum nigrum L. exhibiting significant anti-cancer activity (Table 1) [176]. Solanine reduced the TGF-β and inhibited the activation of the TGF-β/Smad signaling pathway to stop the progression of HCC. The TGF-β/Smad signaling pathway was associated with the proliferation and differentiation of immunosuppressive cells. Therefore, the proportion of CD4+CD25+Foxp3+ Tregs in the TME was reduced in the detection [122]. Moreover, the infiltration of IL-10 was also inhibited in the solanine-treated groups [122]. With the reprogramming of TIME in the HCC, the anti-cancer efficiency of immune response was recovered by the solanine, embodying in smaller size and a lower rate of metastasis of the hepatic tumor [122]. Recently, Yin S et al. reported that solamargine (a derivative of solanine)’ s function of targeting the TIME in the HCC-bearing mice [123]. Solamargine initiated the repolarization of M2-like towards M1-like phenotype via decreasing the expression of leukemia inhibitory factor (LIF) derived from tumor cells, an immunosuppressive mediator that activated the STAT3 signaling to alter the differentiation of immune cells [123,177]. Additionally, the treatment of solamargine induced the activation and recruitment of DCs and the decrease of MDSCs in the TME. The enhanced infiltration of CD4+ T cells was observed, indicating that the recovered immune response was achieved in the HCC models after treatment with the solamargine [123].

#### 3.7.3. Terpene Glycosides

##### Astragaloside IV

Astragaloside IV, a tetracyclic triterpenoid saponin, was isolated from the astragalus polysacharin complex and seemed a quality-control indicator of the Radix Astragali in both Chinese and European Pharmacopoeia (Table 1) [178]. Li C et al. first discovered that M2-like TAMs promoted the proliferation and migration of hepatic tumor cells, which was suppressed by the astragalus polysacharin [179]. This result inspired the researcher to deeply explore which components in the astragalus polysacharin impact the M2-like polarization of TAMs. Soon after, Min L et al. reported the potential role of astragaloside IV in manipulating the polarization of TAMs in the TIME of HCC [124]. Astragaloside IV blocked the polarization of TAMs towards M2-like phenotypes via suppressing the activation of the TLR4/NF-κB/STAT3 signaling pathway, thus recovering the innate immunity to inhibit the proliferation of HCC cells [124].

##### Paeoniflorin

Paeoniflorin is a natural monoterpenoid glycoside extracted from multiple herbs, such as Paeonia lactiflora Pall. and Cimicifuga foetida L. etc., (Table 1). The studies illustrated that PD-L1 in HepG2 was significantly reduced after treatment with 20 μM paeoniflorin for 24h [125]. Furthermore, the researcher discovered that paeoniflorin could inhibit the phosphorylation of both JAK and STAT3 pathways, which were associated with the expression of PD-L1 in tumor cells. Following the inhibition of the immunosuppressive microenvironment, the activities of effector T cells were also enhanced, and more IL-2 secretion was detected [125].

##### Gastrodin

Gastrodin, a phenolic glycoside, exists abundantly in Gastrodia elata Blume which is considered to be a famous restorative food in some areas of China and exhibits immunomodulation activity in previous studies (Table 1) [126]. Shu G et al. concluded that gastrodin could be used as an auxiliary reagent in HCC therapy as they founded that it repressed the tumor cells growth in vivo with low toxicity [126]. The level of IL-4, a representative Th2 cytokine which impaired cytotoxic activities of CD8+ T and NK cells, was downregulated by gastrodin. After gastrodin administration, the reduced percentage of CD4+ T cells was rescued and the cytotoxic activities of CD8+ T cells and NKs were increased, which suggested that the gastrodin alleviated the systematic immune suppressive circumstance and stimulated the naturally anti-cancer immune response in the host [126].

## 4. Conclusions and Perspectives

We want to conclude this long story about the value of natural compounds in HCC treatment with a few critical points. First, single-drug immunotherapy of HCC using natural compounds is efficient and prominent. The effect of reversing the immunosuppression via targeting the TIME is supported by the current literature. Second, the fact that natural compounds work on multiple targets and pathways favors their function in complicated immune system regulation, thus avoiding the low responsivity and frequent relapse deficiency of single-target drugs. Finally, based on the immunoregulatory effects of natural compounds, combined-drug therapy could be a potential strategy in future HCC therapy.

For the treatment of advanced HCC, the conventional therapy with single-target drugs is limited due to inter-individual variability, tumor heterogeneity and migration, and the TME complexity [12]. Moreover, the single-target treatment neglects the correlation of multi-etiologies and underestimates the recalcitrance and adaptability of cancer in response to external stimulation. Therefore, awakening the innate immune is preferable in the HCC treatment due to its advantages of inducing a spontaneous anti-cancer response in patients. Recently, the application of the natural products in immunotherapy for anti-HCC is becoming prominent due to its extensive range of immunomodulatory activities, fewer side effects, and adequate resources [12]. As we discussed, natural compounds, as a kind of natural products, can play a very positive role in targeting TIME to reverse the immunosuppression, such as modulating the generation and polarization of immunosuppressive cells (Tregs, MDSCs, TAMs, etc.) and inhibiting the secretion of immunosuppressive cytokines (IL-6, IL-10, TGF-β, etc.). Moreover, natural compounds contribute to the induction of ICD or the expression of pattern recognition receptors (PRRs) to enhance immunogenicity, further activating the immune response to kill the HCC cells. Due to their distinctive advantages over chemotherapeutic drugs, natural compounds have been extensively explored for their anti-HCC activities in the clinical trials, whatever as drugs or supplements, despite the fact that there are still no approved drugs for HCC therapy. For instance, icaritin we discussed above, showed potential anti-HCC activity and safety profiles in a phase I clinical study, as a potential oral immunotherapy for advanced HCC. Compared with sorafenib, HCC patients treated with icaritin demonstrated a comparable overall response rate but likely with more durable survival benefit and higher tolerability [49]. Moreover, some clinical results also implied that the natural compounds, such as resveratrol and luteolin, as adjuvants could probably prolong median survival time and improve the prognosis of postoperative HCC patients [180,181,182].

Notably, although abundant studies about the efficiency of naturally occurring compounds on anti-HCC therapy have been reported, either as the single-agents or adjuvant agents, there are still many problems regarding the development of natural compounds that need to be solved. First, the pharmaceutical utilization of natural compounds must be standardized. However, an international consensus has not been reached regarding the standards for the origination and production of natural compounds. Our lack of comprehension and solution of their crucial parameters, such as the low content of active compounds, the complicated composition of natural extracts, and numerous coexistences of their related isomers, limits their production/isolation, especially in the large scale. Moreover, the quality of the natural resources is hard to maintain due to the variation of many environmental factors. The variation in concentration of active compounds in the natural resources makes them harder to product, especially by isolation. To improve the quality of natural compounds produced from the nature, we should establish a sophisticated reference standard and strictly control the origin and harvest date of crude drugs. Second, the limitations of many natural compounds, such as poor water solubility, low bioavailability in vivo, and low hepatic distribution, have severely restricted their further clinical application in the HCC therapy, while some potential natural compounds can only be used as supplements to standard HCC therapy [183,184]. These issues could be resolved in the future via improving the drug delivery system that improve outcomes of tumor-targeting, immune-sensitization, stability, and safety of natural compounds in treating HCC [10]. In addition, a further structural modification of these original naturally occurring compounds is also considered for the officinal development. Finally, the immune regulation by biological compounds involves multiple signal transduction pathways, but the mechanism is not fully understood, which brings underlying risks in the clinical application of natural compounds. Regarding the lack of these important information, more delicate scientific studies and clinical trials are needed to examine the safety and efficiency of the potential natural compounds in the HCC therapy. In conclusion, compounds originating from the nature shed new light on developing immune therapy against cancers, especially against HCC. With the rapid evolution of healthy biotechnology and knowledge, natural products will significantly contribute to breakthroughs in HCC therapy.

## Data Availability

Not applicable.

## Abbreviations

HCC	Hepatocellular carcinoma
TIME	Tumor immunosuppressive microenvironment
NAFLD	Nonalcoholic fatty liver disease
ICIs	Immune checkpoint inhibitors
PD-1	Programmed cell death-1
PD-L1	Programmed cell death-ligand 1
CTLA-4	Cytotoxic T-lymphocyte-associated protein 4
TME	Tumor microenvironment
APP	Antigen processing and presentation
APCs	Antigen-presenting cells
DCs	Dendritic cells
KCs	Kupffer cells
IL	Interleukin
Tregs	Regulatory T cells
CTLs	Cytotoxic T lymphocytes
TGF	Transforming growth factor
TAMs	Tumor-associated macrophages
Arg1	Arginase 1
IDO	Indoleamine 2,3-dioxygenase
MDSCs	Marrow-derived suppressor cells
ROS	Reactive oxygen species
iNOS	Inducible nitric oxide synthase
CAFs	Cancer-associated fibroblasts
PGE2	Prostaglandin E2
ECM	Extracellular matrix
ICD	Immunogenic cells death
CRT	Calreticulin
HMGB1	High mobility group protein B1
DAMPs	Damage-associated molecular patterns
IFN	Interferon
EMH	Extramedullary hematopoiesis
BrMC	8-bromo-7-methoxychrysin
CoCl_2_	Cobalt chloride
VEGF	Vascular endothelial growth factor
MMP	Matrix metalloproteinase
HIF	Hypoxia-inducible factor
GrzB	Granzyme B
DHZCP	Dahuang Zhechong pill
Th	T helper
HSCs	Hepatic stellate cells
ER	Endoplasmic reticulum
ATP	Adenosine triphosphate
HSP	Heat-shock protein
TrxR	Thioredoxin reductase
IRE	Inositol-requiring enzyme
XBP	X-box-binding protein
TRAFal	TNF receptor-associated factor
IKK	Inhibitor of κB kinase
TLR	Toll-like receptor
α-SMA	α-smooth muscle actin
CCL	C-C motif chemokine ligand
CXCL	CXC chemokine ligand
TNFR1/TNFRSF1A	Tumor necrosis factor receptor superfamily member 1
KAT	Lysine acetyltransferase
imDCs	Immature DCs
4-MU	4-Methylumbelliferone
HA	Hyaluronic acid
CSCs	Cancer stem cells
SY	Safflower yellow
HSYA	Hydroxyl safflower yellow A
LIF	Leukemia inhibitory factor
PRRs	Pattern recognition receptors
